# The prevalence and nature of cardiac arrhythmias in horses following general anaesthesia and surgery

**DOI:** 10.1186/1751-0147-53-62

**Published:** 2011-11-23

**Authors:** Ruth A Morgan, Alexandra G Raftery, Peter Cripps, Jonathan M Senior, Catherine M McGowan

**Affiliations:** 1 Philip Leverhulme Equine Hospital, University of Liverpool, Leahurst Campus, Neston, Cheshire, CH64 7TE, UK; 2 Institute of Infection and Global Health, University of Liverpool, Leahurst Campus, Neston, Cheshire, CH64 7TE, UK; 3 Institute of Ageing and Chronic Disease, University of Liverpool, Leahurst Campus, Neston, Cheshire, CH64 7TE, UK

**Keywords:** equine, post-anaesthetic, electrocardiography, arrhythmia

## Abstract

**Background:**

The prevalence and nature of arrhythmias in horses following general anaesthesia and surgery is poorly documented. It has been proposed that horses undergoing emergency surgery for gastrointestinal disorders may be at particular risk of developing arrhythmias. Our primary objective was to determine the prevalence and nature of arrhythmias in horses following anaesthesia in a clinical setting and to establish if there was a difference in the prevalence of arrhythmias between horses with and without gastrointestinal disease undergoing surgery. Our secondary objective was to assess selected available risk factors for association with the development of arrhythmias following anaesthesia and surgery.

**Methods:**

Horses with evidence of gastrointestinal disease undergoing an exploratory laparotomy and horses with no evidence of gastrointestinal disease undergoing orthopaedic surgery between September 2009 and January 2011 were recruited prospectively. A telemetric electrocardiogram (ECG) was fitted to each horse following recovery from anaesthesia and left in place for 24 hours. Selected electrolytes were measured before, during and after surgery and data was extracted from clinical records for analysis. Recorded ECGs were analysed and the arrhythmias characterised. Multivariable logistic regression was used to identify risk factors associated with the development of arrhythmias.

**Results:**

Sixty-seven horses with gastrointestinal disease and 37 without gastrointestinal disease were recruited. Arrhythmias were very common during the post-operative period in both groups of horses. Supra-ventricular and bradyarrhythmias predominated in both groups. There were no significant differences in prevalence of any type of arrhythmias between the horses with or without gastrointestinal disease. Post-operative tachycardia and sodium derangements were associated with the development of any type of arrhythmia.

**Conclusions:**

This is the first study to report the prevalence of arrhythmias in horses during the post-operative period in a clinical setting. This study shows that arrhythmias are very common in horses following surgery. It showed no differences between those horses with or without gastrointestinal disease. Arrhythmias occurring in horses during the post-anaesthetic period require further investigation.

## Background

The prevalence of arrhythmias in horses following general anaesthesia is unknown. Texts report that horses undergoing emergency exploratory laparotomy (colic surgery) commonly have arrhythmias in the post-operative period, yet there is very limited literature in this area [1,2]. Arrhythmias have been reported in association with gastrointestinal disease [3]. In one case series of horses with ventricular tachycardia, four out of twenty-one had undergone colic surgery [4]. The reported risk factors associated with the development of arrhythmias include hypoxia, endotoxaemia, electrolyte imbalances and anaesthesia [5]. It seems logical to assume that a horse suffering from gastrointestinal disease will be at greater risk of developing arrhythmias because they may be more often exposed to these factors. If the prevalence and nature of arrhythmias in the post-anaesthetic period is determined, then clinicians will be better able to identify, understand and manage those horses developing post-operative arrhythmias.

In this study we aimed to determine the prevalence and nature of arrhythmias occurring in the post-anaesthetic period in a hospital setting and to determine if there was a difference in the prevalence of arrhythmias between horses with and without gastrointestinal disease undergoing general anaesthesia and surgery. A secondary aim was to assess certain potential risk factors that may be associated with the development of arrhythmias in horses following surgery.

We hypothesised that the prevalence of arrhythmias would be high in horses following surgery, and that the prevalence would be higher in horses with gastrointestinal disease.

## Methods

### Study population

This study was approved by the University of Liverpool ethics committee and informed consent was obtained from all study participants.

Horses with evidence of gastrointestinal disease undergoing exploratory laparotomy and horses without evidence of gastrointestinal disease undergoing orthopaedic surgery at the Philip Leverhulme Equine Hospital, University of Liverpool between September 2009 and January 2011 were eligible for inclusion in the study. Horses were included in the gastrointestinal group (GI) if they had signs of abdominal discomfort (colic) which required surgical exploration and were found to have a lesion of the gastrointestinal tract at surgery. Horses were included in the non-gastrointestinal disease group (non-GI) if they were undergoing orthopaedic surgery, had shown no signs of colic in the previous 24 hours and had normal clinical parameters and faeces prior to surgery. Horses were excluded from the study if they were less than one year old, mares with foals at foot, those that did not tolerate the ECG, were euthanased either during or within 24 hours of recovery from anaesthesia, or were diagnosed with grass sickness or a non-gastrointestinal lesion at surgery. GI group horses were excluded as non-GI group horses if they subsequently underwent orthopaedic surgery and vice versa.

### Data Collection

Clinical data was extracted from the case files of those horses included in the study to obtain details prior to surgery, during surgery and during the post-operative period.

Heparinised blood was collected prior to surgery, immediately after recovery from anaesthesia (T0), 12 hours after recovery (T12) and 24 hours after recovery (T24). The blood was analysed using a blood gas analyzer (Radiometer ABL77, Radiometer) to measure plasma concentrations of potassium, ionized calcium, sodium and chloride. The ABL77 was calibrated each time prior to use if it had not been calibrated in the previous eight hours. Colloid osmotic pressure (COP) was measured using an oncometer (Onkometer BMT 923, Vitech Scientific Ltd). The oncometer underwent a two point calibration every week against water and a standardized solution and a one point calibration prior to every use.

An ECG Holter monitor (Televet 100, Krutech) was fitted to each horse on recovery from anaesthesia, with electrodes placed in a modified base-apex lead configuration. The positive electrode was placed 30 cm below the withers on the left thorax, the negative electrode was placed just to the right of the sternum. Two further electrodes were placed approximately 10 cm below the positive electrode and over the left apex of the heart. The recording device was secured to the horse using a surcingle and anti-cast roller. Telemetric mode was used to ensure the quality of trace after application and during the recording period.

The ECG recordings were stored on a SD card and downloaded into Televet 100 software version 4.2 (Kruuse A/S). The traces were assessed for diagnostic quality. Diagnostic traces were defined as those allowing clear identification of QRS depolarisations for more than 20 hours.

### ECG Analysis

Televet software was used to determine R-R intervals. An R-R interval deviation of 20% was chosen as the maximum accepted deviation as reported in previous studies [6]. Deviations of greater than 20% were detected by the software and then manually analysed by the author (RM) to characterise the arrhythmia.

Arrhythmias were defined as follows:

• Sinus Arrhythmia (SA): a rhythmical variation in R-R intervals in which the heart rate slowed and then increased again. The R-R interval increased by less than 100%.

• Supra-ventricular premature depolarisation (SVPD): The R-R interval decreased by greater than 20%. A P wave (usually of abnormal configuration) visible followed by a QRS complex that was very similar to the preceding QRS complexes. The P wave may not have been visible if the heart rate was very high (> 70 bpm) but the complex was judged to be supra-ventricular if the QRS had the same configuration as those preceding it.

• Ventricular premature depolarisation (VPD): The R-R interval decreased by greater than 20%. No P wave preceding the QRS. A QRS of abnormal configuration.

• Ventricular tachycardia (VT): four or more VPDs occurring in succession.

• Second Degree Atrioventricular block (2AVB): A P wave without a subsequent QRS complex and an increase in R-R interval of 100%.

• Sinus block (SB): An increase in R-R interval of more than 100% with no visible P wave.

• Bradyarrhythmia: Sinus arrhythmias, second degree atrioventricular block and sinus block.

The number of SVPDs and VPDs occurring during the recording period was recorded.

### Statistical Analysis

Data were extracted and entered into an excel spreadsheet. Statistical analysis was carried out in Minitab 16 (Minitab Inc) and STATA for Windows 9 (STATA Corp). Standard descriptive statistics were performed on the data followed by univariable analyses using logistic regression.

To compare the breed, age and sex of the two groups and the prevalence of different arrhythmias between the groups chi-squared analysis and Mann-Whitney *U *tests were performed. To compare the number of SVPDs and VPDs occurring in each group a Mann-Whitney *U *test was used due to the non-parametric nature of the data. Statistical significance was defined as *P *< 0.05.

Explanatory variables included in the logistic regression were: the presence or absence of gastrointestinal disease, age, breed, sex, weight, pre-operative heart rate, pre-operative score assigned to the patient according to the American Society of Anaesthesiologist's physical classification system (ASA) [7], pre-operative plasma concentrations (mmol/L) of potassium, ionised calcium, sodium, chloride and colloid osmotic pressure (mmHg). Intra-operative factors included anaesthetic agent, administration of lidocaine, a period of intra-operative hypoxia (PaO_2 _< 80 mmHg), intra-operative plasma concentrations of potassium, ionised calcium, sodium and chloride. Post-operative factors included heart rate at T0, T12 and T24, plasma concentrations of potassium, ionised calcium, sodium and chloride all at T0, T12 and T24, post-operative fluid and lidocaine administration and survival to discharge.

Outcomes assessed using logistic regression included the presence of SVPDs or VPDs during the recording period and the presence of a bradyarrhythmia which included one or more of the following: sinus arrhythmia, sino-atrial block and second degree atrioventricular block. In order to take into account the wide range of numbers of premature depolarisations occurring during the recording period (0-400 SVPDs per horse), outcomes based on the number of premature depolarisations occurring were also examined. The number of SVPDs occurring in the population was divided into quartiles (Table 1) and assessed as a quartile outcome using ordinal logistic regression. Given the distribution of the frequency of VPDs an outcome of less than or equal to 2 or greater than 2 VPDs during the recording period was used as an outcome and investigated using binary logistic regression.

**Table 1:** The number of premature depolarisations occurring in both groups of patients during the recording period

Frequency	SVPDs (n, %)	VPDs (n, %)
**0-1**	27 (26%)	56 (54%)

**2-4**	27 (26%)	20 (19%)

**5-20**	25 (24%)	19 (18%)

**20-400**	25 (24%)	9 (9%)

All variables which were associated with the outcome with a change in deviance (log-likelihood ratio) at *P *< 0.25 were offered to a forward multivariable logistic regression. The results were then verified using a backward stepwise regression model. The overall fit of the model was ascertained using the Hosmer and Lemeshow Goodness of Fit Test [8]. Criterion for inclusion was a change in deviance at *P *< 0.05.

## Results

### Study Population

One hundred and four horses were included in the study. Of those, 67 were horses with gastrointestinal disease undergoing surgery (GI group) and 37 were horses without gastrointestinal disease undergoing orthopaedic surgery (non-GI group). The types of surgery undertaken in both groups are shown in Table 2.

**Table 2:** Distribution of surgical lesions/procedures

GI group	Number (%) of horses
Strangulating small intestinal	28 (42%)

Non-strangulating small intestinal	7 (11%)

Strangulating large intestinal	10 (15%)

Non-strangulating large intestinal	22 (33%)

**Non-GI group**	

Treatment of synovial sepsis	18 (49%)

Diagnostic arthroscopy/tenoscopy	14 (38%)

Annular ligament desmotomy	2 (5%)

Small tarsal joint arthrodesis	1 (3%)

Splint bone removal	1 (3%)

Neurectomy and fasciotomy	1 (3%)

The mean age of the GI group was 11.5 years (1-30 years). Twenty nine (43%) were mares, 37 (55%) were geldings and one (2%) was a stallion. The breeds represented in this group were 16 (24%) Cobs, 16 (24%) Thoroughbreds, 12 (18%) Warmbloods, four (6%) Welsh ponies, eight (12%) Irish drafts and 11 (16%) were other breeds. The mean age for the non-GI group was 11 years (4-21 years). Twenty-six (70%) were geldings and 11 (30%) were mares. The breeds represented in the non-GI group included three (8%) Cobs, 15 (41%) Thoroughbreds, three (8%) Warmbloods, six (16%) Welsh ponies and two (5%) Irish Drafts and eight (22%) were other breeds. The groups were not different in age (*P *= 0.61). Breed distribution was different between the groups (*P *= 0.04) with Thoroughbreds overrepresented in the non-GI group.

The pre-operative management of the cases depended on clinical presentation and the type of surgery undertaken and was determined by the clinician and anaesthetist in charge of the case. All horses in the non-GI group were deprived of food for at least 10 hours prior to surgery but they were allowed free access to water. All of the horses in the study received intravenous crystalloids during surgery titrated according to requirements as determined by the anaesthetist. Forty-five (67%) of colic patients received intravenous crystalloids following surgery. Of these all but one received supplementary potassium and calcium. Overall 42 (63%) of the colic patients received lidocaine during the peri-operative period; 40 (62%) during surgery and 20 (30%) during the post-operative period.

Hypokalaemia and hypocalcaemia were common in both groups in the post-operative period (Table 3). In the non-GI group the prevalence of hypokalaemia was significantly lower at T12 (*P *= 0.002) and the prevalence of hypocalcaemia was significantly lower at T12 (*P *< 0.001) and T24 (*P *< 0.001).

**Table 3:** The prevalence of hypokalaemia and hypocalcaemia in patients with and without gastrointestinal disease during the post-anaesthetic period

Time post surgery	0 hours	12 hours	24 hours
n (%) of horses **with GI disease with hypokalaemia **(< 3.5 mmol/l)^a^	38 (58%)	55 (85%)	47 (71%)

n (%) of horses **without GI disease with hypokalaemia**	19 (53%)	21(58%)	21(62%)

n (%) of horses **with GI disease with hypocalcaemia **(< 1.45 mmol/l)^b^	60 (92%)	42 (66%)	28 (48%)

n (%) of horses **without GI disease with hypocalcaemia**	35 (97%)	9 (25%)	2 (6%)

^a ^Borer and Corley 2006 [31]

^b^Van der Kolk et al 2002 [32]

### Prevalence of Arrhythmias

The use of the Telemetric ECG was well tolerated by the study population and produced ECG traces of diagnostic quality.

Arrhythmias were common following surgery in both groups (Table 4), with SVPDs and bradyarrhythmias most commonly detected. Ventricular tachycardia was recorded in only two of the GI group and none of the non-GI group. The presence of VT was not associated with death. There were no statistically significant differences between the GI group and the non-GI groups in the prevalence of SVPDs (*P *= 0.60) or VPDs (*P *= 0.77) but second degree AV block was more prevalent in the non-GI group than in the GI group (*P *= 0.04).

**Table 4:** Prevalence of different arrhythmias during the post-anaesthetic period in horses with and without gastrointestinal disease

Arrhythmia	GI Group N (%) (95% Confidence Interval)	Non-GI Group N (%) (95% Confidence Interval)
**One or more SVPD**	57 (85%) (75%, 93%)	30 (81%) (65%, 92%)

**One or more VPD**	40 (60%) (47%, 72%)	20 (54%) (39%,73%)

**Ventricular Tachycardia**	2(3%) (0.36%, 10.4%)	0 (0%) (0%, 13%)

**Sinus Arrhythmia**	53 (79%) (67%, 88%)	31 (84%) (68%, 94%)

**2^nd ^degree AV block**	24 (36%) (24%, 48%)	21 (57%) (39%, 73%)

**Sino-atrial Block**	3 (5%) (0.9%, 13%)	3(8%) (1.7%, 23%)

SVPD- supraventricular premature depolarisation

VPD - Ventricular premature depolarisation

AV - atrioventricular

The mean number of SVPDs occurring in both groups during the recording period was 27 (0-400) and the mean number of VPDs occurring in both groups was 7.5 (0-161). The mean number of SVPDs in the GI group was 30 (0-400) and in the non-GI group was 21 (0-400), with no difference between groups (*P *= 0.09). The mean number of VPDs in the GI group was 7 (0-100) and in the non-GI group was 9 (0-161), with no difference between groups (*P *= 0.57). The distribution of arrhythmia frequency is shown in Table 5.

**Table 5:** Multivariable ordinal logistic regression model of risk factors for developing SVPDs during the post-anaesthetic period

Predictor	OR	95% CI	P-value
**Post-operative Na T12 (mmol/l)**	0.81	0.71-0.92	0.001*

**ASA score**			
**Reference 1**	1.0		
**2**	0.53	0.17-1.65	0.273
**3**	0.20	0.07-0.60	0.004*
**4**	0.23	0.08-0.65	0.006*
**5**	1.60	0.10-26.95	0.743

Log-Likelihood = -125.765

Test that all slopes are zero: G = 20.063, DF = 5, P-Value = 0.001

### Identification of risk factors associated with development of arrhythmias following surgery

The variables which were significant after univariable analysis are given in the additional files for each of the five outcomes; 1 or more SVPD (Additional file 1 Additional File 2), SVPDs in quartiles (Additional File 3 Additional File 4), 1 or more VPD (Additional File 5 Additional File 6), greater than 2 VPDs (Additional File 7 Additional File 8), bradyarrhythmias (Additional File 9 Additional File 10).

Multivariable analysis showed an association between higher sodium concentration at T12 and the likelihood of developing SVPDs). Ordinal regression was used to investigate risk factors which may be associated with the number of SVPDs developing. In the final model a higher sodium concentration at T12 and a higher ASA score resulted in an overall decreased likelihood of having a higher number of SVPDs (Table 5). The presence or absence of gastrointestinal disease was significant in the model but was not retained due to the high collinearity of this factor with ASA score (*P *< 0.001).

The risk of more than one VPD in the recording period was increased by high heart rate at T12 and higher sodium at T24 (Table 6). When the outcome of more than 2 VPDs was investigated in a multi-variable model, no risk factors were identified. In contrast, higher heart rates at T0 decreased the likelihood of developing bradyarrhythmias.

**Table 6:** Multivariable binary logistic regression model of risk factors for the development of VPDs during the post-anaesthetic period

Predictor	OR	95% CI	P-value
HR T12 (bpm)	1.03	1.00-1.07	0.042*

Post-operative Na T24 (mmol/l)	1.19	1.03-1.38	0.021*

Log-Likelihood = -62.88

Test that all slopes are zero: G = 9.203, DF = 2, P-Value = 0.01

## Discussion

This is the first study to report the prevalence of different arrhythmias in horses during the post-anaesthetic period and has shown that cardiac arrhythmias occur in a very high proportion of horses following anaesthesia. Few of the risk factors investigated were found to be associated with the presence of absence or SVPDs and VPDs.

It is known that physiological arrhythmias are common in healthy horses at rest. Studies have shown that sinus arrhythmia, second degree atrioventricular block and isolated premature depolarisations occur at rest in the normal horse [9,10]. In horses, sinus arrhythmia has been most commonly detected during the recovery period following exercise and is considered a manifestation of the switch from dominance of the sympathetic drive to parasympathetic drive [11]. Sinus arrhythmia was noted in 81% of horses in this study population; it is plausible that during the post-operative period a similar change in autonomic balance occurs. As expected, the development of bradyarrhythmias (SA, 2AVB and SB) was less likely in the presence of a higher heart rate in this population. No other risk factors were identified.

SVPDs and VPDs were more common in the population in this study than previously reported in healthy horses at rest and at exercise. Supra or ventricular depolarisations may be associated with underlying cardiac or non cardiac disease, but they have also been demonstrated in normal animals. Infrequent SVPDs have been shown to occur at rest, during warm-up, exercise and recovery in 22.4% to 76.2% of clinically healthy horses [6,12,13]. VPDs occur less frequently in the resting horse, with prevalence reports of between 0% [6] and 3.7% [13]. Dogs, cats and humans all have a high prevalence (32-78%) of VPDs during 24 hour ECG recordings of apparently healthy subjects [14-17]. In this study SVPDs were more common than VPDs but both occurred in a large proportion of horses with and without gastrointestinal disease.

There were no statistically significant differences in the prevalence of SVPDs or VPDs in horses with or without gastrointestinal disease in this study. The presence of gastrointestinal disease (reflected in a higher ASA score) was associated with a reduction in the likelihood of developing a high number of SVPDs and was not a risk factor for developing VPDs. These findings differ from previous literature [2]. In one study of horses undergoing surgery [2], eight out of 35 horses undergoing colic surgery developed more than one VPD per hour; four developed VT and 11 had more than one SVPD per hour during the post-operative period. However, none of the horses undergoing orthopaedic surgery developed a tachyarrhythmia [2]. In contrast, in the study presented here, the majority of horses had an arrhythmia and most had one or more SVPD during the post-operative period. Fewer horses developed VPDs and only two had periods of VT, both of these latter horses being in the GI group. It is, however, difficult to compare the two studies as the criteria for defining arrhythmias and the methods of recording them were different. Furthermore, based on the prevalence reported in that study [2], our study only had a power of 0.6 for 95% confidence of detecting a difference between the groups despite achieving twice the numbers in each group. It would appear from our data that the presence or absence of gastrointestinal disease is not as important as the act of general anaesthesia and surgery in the development of arrhythmias. However, based on the data collected in this study, a retrospective power calculation shows that in order to detect a difference in, for example the presence of VPDs, between the populations over 2000 horses would be required. We would suggest a further multi-centred study may be required to demonstrate such a difference.

There is little published data on the prevalence of post-operative arrhythmias in human medicine, but routine electrocardiographic monitoring is carried out in most recovery rooms. The American Society of Anaesthesiologists (ASA) guidelines [18] for post-operative monitoring state that 'The literature is insufficient to evaluate the impact of cardiovascular assessment and monitoring on peri-operative complications, and the literature is silent regarding routine electrocardiographic monitoring.' A recent review of the human literature [19] found an overall prevalence rate for post-operative arrhythmias of 7% but noted the lack of large scale prospective studies using continuous monitoring. Cardiac arrhythmias in dogs following surgery are common; Buhl et al. [20] showed that 93% of dogs had a pathological arrhythmia either during the first 24 hours following surgery or during a 24 hour period 5 days after surgery.

Proposed risk factors for the development of arrhythmias include endotoxaemia, electrolyte disorders, acidosis, hypoxia or severe pulmonary disease, administration of drugs and anaesthesia [4]. In this study, a limited number of these factors were investigated and, of these, very few were associated with the development of the different types of arrhythmia. Sodium appeared to influence the development of SVPDs and VPDs; this may reflect the hydration status of the horses following surgery and would require further investigation. A higher ASA score in this study was found to reduce the likelihood of having a higher number of SVPDs. The reasons for this are unclear but one could speculate that horses assessed pre-operatively to be in a worse physiological condition may receive different post-operative care and may have altered autonomic activity compared to their healthier counterparts. The horse's pre and post-operative requirements for analgesia and other drugs, such as alpha 2 agonists, also require consideration. Further investigation of factors which have not been included in the current study, such as packed cell volume, total protein, acid-base balance and post-operative management that may be associated with the development of arrhythmias is warranted.

Abnormalities of plasma electrolyte concentrations are frequently cited as a cause of cardiac arrhythmias. Horses with gastrointestinal disease often have significant electrolyte imbalances the most common of which are hypokalaemia, hypomagnesaemia and hypocalcaemia [21,22]. Hypocalcaemia and hypokalaemia were common in horses both with and without gastrointestinal disease following surgery in this study and yet plasma electrolyte concentrations were not associated with the development of either SVPDs or VPDs. In common with other equine hospitals, our patients receive intensive monitoring and, if necessary, intravenous supplementation of electrolytes. Consequently none of the horses in this study had profound electrolyte abnormalities. It is unknown whether more pronounced electrolyte abnormalities may have induced further arrhythmias.

Studies have shown that the majority of horses undergoing colic surgery have a degree of hypomagnesaemia [22]. It is unfortunate that due to the constraints of the clinic facilities we were unable to measure magnesium in this study. The direct association between low magnesium levels and arrhythmias is unclear [23] and further work in this area is warranted.

Endotoxaemia has been associated with the development of arrhythmias in horses with gastro-intestinal disease. Possible mechanisms for this include ischaemic injury to the myocardium, electrolyte imbalances and alterations in membrane permeability. Reported markers of endotoxaemia include pyrexia, tachycardia, tachypnoea, alterations in mucous membrane colour, neutropaenia and elevation in packed cell volume and total protein [24]. In this study specific markers of endotoxaemia were not investigated. It can be assumed that horses with gastrointestinal disease, increased pre-operative heart rate, post-operative heart rate and those requiring post-operative intravenous fluid therapy may be more likely to be suffering from endotoxaemia than others. Of these only an increased heart rate at T12 increased the risk of development of VPDs. Further investigation of endotoxaemia markers for an association with arrhythmias is warranted, but given the similarities in arrhythmia prevalence between the non-GI and GI groups it is unlikely that the presence of endotoxaemia had a profound effect on the development of arrhythmias in this population. The measurement of cardiac troponin 1 may have been a useful addition to determine if there was extensive myocardial damage in any of the horses following anaesthesia and surgery [25].

The findings of this study indicate that rather than a primary disease predisposing horses to post-operative arrhythmias, it may be general anaesthesia, surgery and recovery. If this is the case then changes in autonomic balance driven by stress and general anaesthesia may play the larger role in development of cardiac arrhythmias than disease factors such as hypoxia or endotoxaemia. In people, mental and physical stress can increase cardiac electrical instability and a recognised link exists between emotional and physical stressors and spontaneous ventricular arrhythmias [26]. The role of the autonomic nervous system in the development of arrhythmias is an area of increasing interest in human medicine [27,28]. Given the importance of vagal tone in physiological arrhythmias in the horse, it is certainly worth considering an autonomic basis for arrhythmias following surgery. Assessment of heart rate variability may be a useful tool in identifying and quantifying the degree to which autonomic influences affect the likelihood of a horse developing a post-operative arrhythmia [29]. Surgery itself also incurs an inflammatory response which may be responsible for the development of arrhythmias. Further investigation and assessment of hospitalised horses not undergoing surgery is required to ascertain the role of the autonomic nervous system in the development of arrhythmias.

The horses were clinical cases and as such received a number of drugs that may have been anti or pro-arrhythmogenic. The most obvious of these, lidocaine, was administered to 62% of patients with GI disease as is common practice in many referral hospitals. Lidocaine is often used as an anti-arrhythmogenic for treatment of VT in horses [30]. Lidocaine administration, however, did not have a significant influence on the multi-variable models for any of the arrhythmias. The administration of lidocaine may have influenced the results of this study in that it may have reduced the prevalence of arrhythmias within the GI group. This influence would require further investigation but this study gives useful information for clinical cases where lidocaine is used frequently.

The horses undergoing surgery included in this study had survived for at least 24 hours post-operatively. Given these criteria, it is possible that the prevalence of arrhythmias in those horses that do not survive may be different and potentially more clinically significant because these may represent the most critically ill group. Further work is required to ascertain the prevalence and nature of arrhythmias in those horses that fail to survive the immediate post-operative period.

## Conclusions

This study has shown that arrhythmias are common in this clinical population of horses following surgery. SVPDs and bradyarrhythmias such as sinus arrhythmia, AV block and sinus block predominate. The presence of gastrointestinal disease, in these clinical cases which received intensive supportive therapy, did not affect the prevalence of any type of arrhythmia. Post-operative tachycardia and sodium derangements were found to be associated with the development of arrhythmias following surgery, the biological significance of which is unclear. It is possible, therefore, that other factors, such as autonomic balance, also play a role in arrhythmogenesis in the post-operative period. Further, more extensive, investigation of possible risk factors is required to understand the pathogenesis of post-operative arrhythmias. None of the arrhythmias identified in this study required specific treatment and none was directly associated with the death or euthanasia of a case. It is important to be aware of such a high prevalence of arrhythmias during this period and, if tachycardia persists or an abnormal rhythm is identified, further investigation is required.

## Competing interest Statement

The authors declare that they have no competing interests.

## Authors' contributions

RM, AR and JMS initiated and designed the study and collected the data. RM analysed the data. RM and PC performed the statistical analysis. RM, JMS and CM drafted the manuscript. All authors read and approved the final manuscript.

## Authors' Information

**RM **MA VetMB CertAVP (EM) MRCVS Horse Trust Senior Clinical Scholar in Equine Internal Medicine, University of Liverpool

**AR **MA VetMB MRCVS Assistant Veterinary Surgeon

**JMS **PhD BVSc CertVA DipECVAA MRCVS Lecturer in Veterinary Anaesthesia, Head of Veterinary Anesthesia, University of Liverpool

**PC **BVSc BSc, MSc, PhD MRCVS Senior Lecturer in Epidemiology, University of Liverpool

**CM **BVSc DipVetClinStud MACVSc PhD DEIM DipECEIM FHEA MRCVS Senior Lecturer in Equine Internal Medicine, University of Liverpool

## Supplementary Material

Additional file 1 **Outcome Binary SVPD Univariable Categorical Analyses**.docx. Click here for file

Additional file 2 **Outcome Binary SVPD Univariable Continuous Analyses**.docx. Click here for file

Additional file 3 **Outcome Ordinal SVPD Univariable Categorical Analyses**.docx. Click here for file

Additional file 4 **Outcome Ordinal SVPD Univariable Continuous Analyses**.docx. Click here for file

Additional file 5 **Outcome Binary VPD Univariable Categorical Analyses**.docx. Click here for file

Additional file 6 **Outcome Binary VPD Univariable Continuous Analyses**.docx. Click here for file

Additional file 7 **Outcome 2 VPDs Univariable Categorical Analyses**.docx. Click here for file

Additional file 8 **Outcome 2 VPDs Univariable Continuous Analyses**.docx. Click here for file

Additional file 9 **Outcome bradyarrhythmias Univariable Categorical Analyses**.docx. Click here for file

Additional file 10 **Outcome bradyarrhythmias Univariable Continuous Analyses.docx**. Click here for file
