# Supplementary material for: The prevalence and nature of cardiac arrhythmias in horses following general anaesthesia and surgery
Source: Acta Vet Scand. 2011 Nov 23;53(1):62. doi: 10.1186/1751-0147-53-62 (PMC3269988; doi:10.1186/1751-0147-53-62)
Supplement: Additional file 1 — Outcome Binary SVPD Univariable Categorical Analyses.docx. [file 1751-0147-53-62-S1.DOC]

| **Variable**  Univariable binary logistic regression analyses of the categorical variables investigated in the study for their association with one or more **supraventricular premature depolarisation**.  SVPD | **Category** | **Odds Ratio** | **95%Confidence Interval** | **P value** |
| --- | --- | --- | --- | --- |
| **Breed** |  |  |  |  |
| Reference | Cobs | 1.0 |  | 0.03* |
|  | TB | 0.56 | 0.15-2.13 |  |
|  | WB | 3.73 | 0.37-37.58 |  |
|  | Welsh | 8.81 | 0.00-* |  |
|  | ID | 2.40 | 0.23-24.96 |  |
|  | Other | 4.80 | 0.48-47.68 |  |
| **Sex** |  |  |  |  |
| Reference | Male | 1.0 |  |  |
|  | Female | 0.85 | 0.29-2.51 | 0.77 |
| **Anaesthetic Agent** |  |  |  |  |
| Reference | Sevoflurane | 1.0 |  | 0.95 |
|  | Isoflurane | 0.85 | 0.24-3.04 |  |
|  | Halothane | 1.13 | 0.22-5.85 |  |
| **Intra-operative Lidocaine** |  |  |  |  |
| Reference | Yes | 1.0 |  |  |
|  | No | 0.91 | 0.31-2.62 | 0.86 |
| **Period of Hypoxia** |  |  |  |  |
| Reference | Yes | 1.0 |  |  |
|  | No | 1.54 | 0.32-7.52 | 0.58 |
| **Post-operative Lidocaine** |  |  |  |  |
| Reference | Yes | 1.0 |  |  |
|  | No | 0.35 | 0.11-1.11 | 0.08* |
| **Type of Surgery** |  |  |  |  |
| Reference | Colic Surgery | 1.0 |  |  |
|  | Orthopaedic Surgery | 1.33 | 0.46-3.85 | 0.60 |
| **Post-operative Fluids** |  |  |  |  |
| Reference | Yes | 1.0 |  |  |
|  | No | 0.66 | 0.23-1.87 | 0.43 |
| **ASA Score** |  |  |  |  |
| Reference | 1 | 1.0 |  | 0.74 |
|  | 2 | 0.71 | 0.14-3.63 |  |
|  | 3 | 0.63 | 0.13-3.01 |  |
|  | 4 | 1.11 | 0.22-5.51 |  |
|  | 5 | 0.16 | 0.01-3.26 |  |
| **Survival** |  |  |  |  |
| Reference | Yes | 1.0 |  |  |
|  | No | 1.75 | 0.49-6.21 | 0.40 |
