# Supplementary material for: The prevalence and nature of cardiac arrhythmias in horses following general anaesthesia and surgery
Source: Acta Vet Scand. 2011 Nov 23;53(1):62. doi: 10.1186/1751-0147-53-62 (PMC3269988; doi:10.1186/1751-0147-53-62)
Supplement: Additional file 2 — Outcome Binary SVPD Univariable Continuous Analyses.docx. [file 1751-0147-53-62-S2.DOCX]

| **Continuous Variables**  Univariable binary logistic regression analyses of the continuous variables investigated in the study for their association with **supraventricular premature depolarisations**. | **Odds Ratio** | **95%Confidence Interval** | **P value** |
| --- | --- | --- | --- |
| **Age (years)** | 1.03 | 0.93-1.14 | 0.58 |
| **Weight (Kg)** | 1 | 1-1.01 | 0.90 |
| **Pre-operative Heart rate (bpm)** | 0.99 | 0.96-1.02 | 0.51 |
| **Pre-operative Na (mmol/l)** | 0.96 | 0.77-1.19 | 0.71 |
| **Pre-operative K (mmol/l)** | 2.44 | 0.57-10.34 | 0.21* |
| **Pre-operative Ca (mmol/l)** | 4.14 | 0.11-157.85 | 0.45 |
| **Pre-operative Cl(mmol/l)** | 0.95 | 0.82-1.11 | 0.54 |
| **Pre-operative COP (mmHg)** | 1 | 0.81-1.23 | 0.99 |
| **Post-operative Na T0(mmol/l)** | 1.11 | 0.95-1.31 | 0.18* |
| **Post-operative K T0(mmol/l)** | 1.61 | 0.48-5.42 | 0.44 |
| **Post-operative Ca T0(mmol/l)** | 10.7 | 0.18-647.45 | 0.25* |
| **Post-operative Cl T0(mmol/l)** | 1.06 | 0.93-1.19 | 0.38 |
| **Post-operative COP T0 (mmHg)** | 0.92 | 0.74-1.15 | 0.49 |
| **Post-operative Na T12(mmol/l)** | 1.25 | 1.04-1.50 | 0.01* |
| **Post-operative K T12(mmol/l)** | 2.06 | 0.58-7.28 | 0.25 |
| **Post-operative Ca T12(mmol/l)** | 1.07 | 0.02-49.34 | 0.97 |
| **Post-operative Cl T12(mmol/l)** | 1.17 | 1.01-1.37 | 0.03* |
| **Post-operative COP T12 (mmHg)** | 1.03 | 0.89-1.19 | 0.71 |
| **Post-operative Na T24(mmol/l)** | 1.13 | 0.95-1.35 | 0.16* |
| **Post-operative K T24(mmol/l)** | 0.76 | 0.19-2.99 | 0.69 |
| **Post-operative Ca T24(mmol/l)** | 260.91 | 2.38-28545.80 | 0.01* |
| **Post-operative Cl T24(mmol/l)** | 1.18 | 0.99-1.41 | 0.06* |
| **Post-operative COP T24 (mmHg)** | 1.08 | 0.93-1.24 | 0.31 |
| **Post-operative HR0 (bpm)** | 0.98 | 0.95-1.01 | 0.27 |
| **Post-operative HR12 (bpm)** | 0.98 | 0.95-1.01 | 0.25 |
| **Post-operative HR24 (bpm)** | 0.99 | 0.96-1.03 | 0.61 |
