# Supplementary material for: The prevalence and nature of cardiac arrhythmias in horses following general anaesthesia and surgery
Source: Acta Vet Scand. 2011 Nov 23;53(1):62. doi: 10.1186/1751-0147-53-62 (PMC3269988; doi:10.1186/1751-0147-53-62)
Supplement: Additional file 3 — Outcome Ordinal SVPD Univariable Categorical Analyses.docx. [file 1751-0147-53-62-S3.DOC]

| **Variable**  Univariate ordinal logistic regression analyses of the categorical variables investigated in the study for their association with **supraventricular premature depolarisations**.  SVPD | **Category** | **Odds Ratio** | **95%Confidence Interval** | **P value** |
| --- | --- | --- | --- | --- |
| **Breed** |  |  |  |  |
| Reference | Cobs | 1.0 |  |  |
|  | TB | 2.51 | 0.89-7.08 |  |
|  | WB | 1.87 | 0.55-6.34 |  |
|  | Welsh | 0.74 | 0.19-2.93 |  |
|  | ID | 0.79 | 0.20-3.14 |  |
|  | Other | 0.88 | 0.28-2.77 | 0.19* |
| **Sex** |  |  |  |  |
| Reference | Male | 1.0 |  |  |
|  | Female | 1.59 | 0.78-3.24 | 0.20* |
| **Anaesthetic Agent** |  |  |  |  |
| Reference | Sevoflurane | 1.0 |  |  |
|  | Isoflurane | 1.14 | 0.48-2.71 |  |
|  | Halothane | 2.02 | 0.70-5.79 | 0.45 |
| **Intra-operative Lidocaine** |  |  |  |  |
| Reference | Yes | 1.0 |  |  |
|  | No | 0.69 | 0.34-1.42 | 0.32* |
| **Period of Hypoxia** |  |  |  |  |
| Reference | Yes | 1.0 |  |  |
|  | No | 0.74 | 0.28-1.92 | 0.53 |
| **Post-operative Lidocaine** |  |  |  |  |
| Reference | Yes | 1.0 |  |  |
|  | No | 1.17 | 0.49-2.80 | 0.73 |
| **Type of Surgery** |  |  |  |  |
| Reference | Colic Surgery | 1.0 |  |  |
|  | Orthopaedic Surgery | 0.48 | 0.23-1.01 | 0.05* |
| **Post-operative Fluids** |  |  |  |  |
| Reference | Yes | 1.0 |  |  |
|  | No | 0.77 | 0.38-1.53 | 0.45 |
| **ASA Score** |  |  |  |  |
| Reference | 1 | 1.0 |  |  |
|  | 2 | 0.56 | 0.19-1.63 |  |
|  | 3 | 0.35 | 0.12-1.00 |  |
|  | 4 | 0.34 | 0.13-0.92 |  |
|  | 5 | 2.14 | 0.13-36.05 | 0.14* |
| **Survival to discharge** |  |  |  |  |
| Reference | Yes | 1.0 |  |  |
|  | No | 0.60 | 0.24-1.54 | 0.31 |
