# Supplementary material for: The prevalence and nature of cardiac arrhythmias in horses following general anaesthesia and surgery
Source: Acta Vet Scand. 2011 Nov 23;53(1):62. doi: 10.1186/1751-0147-53-62 (PMC3269988; doi:10.1186/1751-0147-53-62)
Supplement: Additional file 4 — Outcome Ordinal SVPD Univariable Continuous Analyses.docx. [file 1751-0147-53-62-S4.DOC]

| **Variable**  Univariable logistic regression analyses of the categorical variables investigated in the study for their association with **ventricular premature depolarisations**.  SVPD | **Category** | **Odds Ratio** | **95% Confidence Interval** | **P value** |
| --- | --- | --- | --- | --- |
| **Breed** |  |  |  |  |
| Reference | Cobs | 1.0 |  | 0.17* |
|  | TB | 1.22 | 0.37-4.06 |  |
|  | WB | 0.21 | 0.05-0.93 |  |
|  | Welsh | 0.87 | 0.18-4.21 |  |
|  | ID | 0.87 | 0.18-4.21 |  |
|  | Other | 1.00 | 0.27-3.74 |  |
| **Sex** |  |  |  |  |
| Reference | Male | 1.0 |  |  |
|  | Female | 0.77 | 0.34-1.73 | 0.53 |
| **Anaesthetic Agent** |  |  |  |  |
| Reference | Sevoflurane | 1.0 |  |  |
|  | Isoflurane | 0.95 | 0.35-2.55 |  |
|  | Halothane | 0.24 | 0.15-1.59 | 0.49 |
| **Intra-operative Lidocaine** |  |  |  |  |
| Reference | Yes | 1.0 |  |  |
|  | No | 1.63 | 0.72-3.70 | 0.24* |
| **Period of hypoxia** |  |  |  |  |
| Reference | Yes | 1.0 |  |  |
|  | No | 1.60 | 0.51-5.03 | 0.41 |
| **Post-operative Lidocaine** |  |  |  |  |
| Reference | Yes | 1.0 |  |  |
|  | No | 1.07 | 0.40-2.90 | 0.89 |
| **Type of Surgery** |  |  |  |  |
| Reference | Colic Surgery | 1.0 |  |  |
|  | Orthopaedic Surgery | 1.13 | 0.50-2.55 | 0.77 |
| **Post-operative Fluids** |  |  |  |  |
| Reference | Yes | 1.0 |  |  |
|  | No | 1.63 | 0.74-3.62 | 0.23* |
| **ASA Score** |  |  |  |  |
| Reference | 1 | 1.0 |  | 0.77 |
|  | 2 | 1.20 | 0.37-3.92 |  |
|  | 3 | 2.12 | 0.65-6.95 |  |
|  | 4 | 1.46 | 0.49-4.36 |  |
|  | 5 | 1.00 | 0.06-18.08 |  |
| **Survival** |  |  |  |  |
| Reference | Yes | 1.0 |  |  |
|  | No | 0.99 | 0.34-2.85 | 0.99 |
