# Supplementary material for: The prevalence and nature of cardiac arrhythmias in horses following general anaesthesia and surgery
Source: Acta Vet Scand. 2011 Nov 23;53(1):62. doi: 10.1186/1751-0147-53-62 (PMC3269988; doi:10.1186/1751-0147-53-62)
Supplement: Additional file 5 — Outcome Binary VPD Univariable Categorical Analyses.docx. [file 1751-0147-53-62-S5.DOCX]

| **Continuous Variables**  Univariable binary logistic regression analyses of the continuous variables investigated in the study for their association with **ventricular premature depolarisations**. | **Odds Ratio** | **95%Confidence Interval** | **P value** |
| --- | --- | --- | --- |
| **Age (years)** | 1.06 | 0.98-1.15 | 0.13* |
| **Weight (Kg)** | 1.00 | 0.99-1.00 | 0.33 |
| **Pre-operative heart rate (bpm)** | 1.00 | 0.98-1.03 | 0.90 |
| **Pre-operative Na (mmol/l)** | 1.07 | 0.92-1.25 | 0.36 |
| **Pre-operative K (mmol/l)** | 0.65 | 0.26-1.61 | 0.35 |
| **Pre-operative Ca (mmol/l)** | 0.51 | 0.04-6.85 | 0.61 |
| **Pre-operative Cl(mmol/l)** | 1.02 | 0.92-1.14 | 0.69 |
| **Pre-operative COP (mmHg)** | 0.94 | 0.81-1.09 | 0.44 |
| **Post-operative Na T0(mmol/l)** | 1.02 | 0.91-1.14 | 0.73 |
| **Post-operative K T0(mmol/l)** | 1.00 | 0.41-2.40 | 0.99 |
| **Post-operative Ca T0(mmol/l)** | 0.45 | 0.02-8.62 | 0.60 |
| **Post-operative Cl T0(mmol/l)** | 1.02 | 0.93-1.11 | 0.68 |
| **Post-operative COP T0 (mmHg)** | 1.03 | 0.87-1.21 | 0.75 |
| **Post-operative Na T12(mmol/l)** | 1.03 | 0.91-1.17 | 0.69 |
| **Post-operative K T12(mmol/l)** | 0.78 | 0.30-1.99 | 0.60 |
| **Post-operative Ca T12(mmol/l)** | 0.77 | 0.04-14.47 | 0.86 |
| **Post-operative Cl T12(mmol/l)** | 0.98 | 0.88-1.09 | 0.67 |
| **Post-operative COP T12 (mmHg)** | 0.94 | 0.84-1.06 | 0.32 |
| **Post-operative Na T24(mmol/l)** | 1.15 | 1.01-1.32 | 0.04* |
| **Post-operative K T24(mmol/l)** | 0.37 | 0.13-1.11 | 0.07* |
| **Post-operative Ca T24(mmol/l)** | 0.36 | 0.01-11.12 | 0.56 |
| **Post-operative Cl T24(mmol/l)** | 1.02 | 0.89-1.16 | 0.82 |
| **Post-operative COP T24 (mmHg)** | 0.93 | 0.83-1.04 | 0.22* |
| **Post-operative HR0 (bpm)** | 1.01 | 0.99-1.03 | 0.42 |
| **Post-operative HR12 (bpm)** | 1.03 | 1.00-1.06 | 0.04* |
| **Post-operative HR24 (bpm)** | 1.03 | 1.00-1.07 | 0.04* |
