# Supplementary material for: The prevalence and nature of cardiac arrhythmias in horses following general anaesthesia and surgery
Source: Acta Vet Scand. 2011 Nov 23;53(1):62. doi: 10.1186/1751-0147-53-62 (PMC3269988; doi:10.1186/1751-0147-53-62)
Supplement: Additional file 6 — Outcome Binary VPD Univariable Continuous Analyses.docx. [file 1751-0147-53-62-S6.DOC]

| Variable  Univariable binary logistic regression analyses to investigate the factors associated with the development of **more than 2 ventricular premature depolarisations.** | Category | Odds Ratio | 95% Confidence Interval | P value |
| --- | --- | --- | --- | --- |
| **Breed** |  |  |  |  |
| Reference | Cobs | 1.0 |  |  |
|  | TB | 1.04 | 0.33-3.27 |  |
|  | WB | 0.40 | 0.09-1.73 |  |
|  | Welsh | 0.74 | 0.16-3.50 |  |
|  | ID | 1.11 | 0.24-5.14 |  |
|  | Other | 1.53 | 0.42-5.50 | 0.59 |
| **Sex** |  |  |  |  |
| Reference | Male | 1.0 |  |  |
|  | Female | 0.56 | 0.25-1.24 | 0.15* |
| **Anaesthetic Agent** |  |  |  |  |
| Reference | Sevoflurane | 1.0 |  |  |
|  | Isoflurane | 0.63 | 0.24-1.68 |  |
|  | Halothane | 0.25 | 0.06-0.97 | 0.086* |
| **Intra-operative Lidocaine** |  |  |  |  |
| Reference | Yes | 1.0 |  |  |
|  | No | 2.29 | 1.02-5.16 | 0.043* |
| Period of Hypoxia |  |  |  |  |
| Reference | Yes | 1.0 |  |  |
|  | No | 1.19 | 0.27-5.14 | 0.82 |
| **Post-operative Lidocaine** |  |  |  |  |
| Reference | Yes | 1.0 |  |  |
|  | No | 1.55 | 0.58-4.14 | 0.38 |
| **Post-operative Fluids** |  |  |  |  |
| Reference | Yes | 1.0 |  |  |
|  | No | 1.81 | 0.83-3.96 | 0.14* |
| **Type of Surgery** |  |  |  |  |
| Reference | Colic | 1.0 |  |  |
|  | Orthopaedic | 1.42 | 0.63-3.21 | 0.40 |
| **ASA score** |  |  |  |  |
| Reference | 1 | 1.0 |  |  |
|  | 2 | 1.48 | 0.43-5.10 |  |
|  | 3 | 2.73 | 0.83-9.01 |  |
|  | 4 | 2.14 | 0.69-6.66 |  |
|  | 5 | 2.14 | 0.21-39.47 | 0.51 |
| **Survival** |  |  |  |  |
| Reference | Yes | 1.0 |  |  |
|  | No | 0.96 | 0.34-2.71 | 0.94 |
