# Supplementary material for: The prevalence and nature of cardiac arrhythmias in horses following general anaesthesia and surgery
Source: Acta Vet Scand. 2011 Nov 23;53(1):62. doi: 10.1186/1751-0147-53-62 (PMC3269988; doi:10.1186/1751-0147-53-62)
Supplement: Additional file 7 — Outcome 2 VPDs Univariable Categorical Analyses.docx. [file 1751-0147-53-62-S7.DOC]

| **Continuous Variables**  Univariable logistic regression analyses of the continuous variables investigated in the study for their association with **development of more than 2 VPDs**. | **Odds Ratio** | **95%Confidence Interval** | **P value** |
| --- | --- | --- | --- |
| **Age (years)** | 1.08 | 1.00-1.17 | 0.05* |
| **Weight (Kg)** | 1.00 | 1.00-1.00 | 0.68 |
| **Pre-operative-op HR (bpm)** | 1.01 | 0.98-1.04 | 0.53 |
| **Pre-operative Na (mmol/l)** | 1.03 | 0.88-1.19 | 0.74 |
| **Pre-operative K (mmol/l)** | 0.72 | 0.29-1.79 | 0.47 |
| **Pre-operative Ca (mmol/l)** | 0.61 | 0.05-8.31 | 0.71 |
| **Pre-operative Cl(mmol/l)** | 0.99 | 0.89-1.11 | 0.89 |
| **Pre-operative COP (mmHg)** | 1.00 | 0.86-1.15 | 0.95 |
| **Post-operative Na T0 (mmol/l)** | 1.00 | 0.90-1.11 | 0.99 |
| **Post-operative K T0 (mmol/l)** | 0.81 | 0.34-1.95 | 0.65 |
| **Post-operative Ca T0 (mmol/l)** | 0.43 | 0.02-7.87 | 0.57 |
| **Post-operative Cl T0 (mmol/l)** | 1.02 | 0.93-1.11 | 0.72 |
| **Post-operative COP T0**  **(mmHg)** | 1.04 | 0.88-1.22 | 0.66 |
| **Post-operative Na T12 (mmol/l)** | 0.98 | 0.86-1.11 | 0.75 |
| **Post-operative K T12 (mmol/l)** | 0.67 | 0.26-1.70 | 0.39 |
| **Post-operative Ca T12 (mmol/l)** | 0.83 | 0.05-14.80 | 0.90 |
| **Post-operative Cl T12 (mmol/l)** | 0.95 | 0.86-1.06 | 0.39 |
| **Post-operative COP T12 (mmHg)** | 0.98 | 0.87-1.10 | 0.71 |
| **Post-operative Na T24 (mmol/l)** | 1.10 | 0.97-1.26 | 0.14* |
| **Post-operative K T24 (mmol/l)** | 0.42 | 0.15-1.21 | 0.10* |
| **Post-operative Ca T24 (mmol/l)** | 0.18 | 0.01-5.50 | 0.32 |
| **Post-operative Cl T24 (mmol/l)** | 1.02 | 0.90-1.17 | 0.73 |
| **Post-operative COP T24 (mmHg)** | 0.94 | 0.84-1.05 | 0.26 |
| **Post-operative HR0 (bpm)** | 1.01 | 0.98-1.03 | 0.65 |
| **Post- operative HR12 (bpm)** | 1.03 | 1.00-1.06 | 0.04* |
| **Post-operative HR24 (bpm)** | 1.03 | 1.00-1.06 | 0.06* |
