# Supplementary material for: The prevalence and nature of cardiac arrhythmias in horses following general anaesthesia and surgery
Source: Acta Vet Scand. 2011 Nov 23;53(1):62. doi: 10.1186/1751-0147-53-62 (PMC3269988; doi:10.1186/1751-0147-53-62)
Supplement: Additional file 8 — Outcome 2 VPDs Univariable Continuous Analyses.docx. [file 1751-0147-53-62-S8.DOC]

| Variable  Univariable binary logistic regression analyses to investigate the factors associated with the development of **sinus arrhythmia, second degree atrioventricular block or sinus bloc**k. | Category | Odds Ratio | 95% Confidence Interval | P Value |
| --- | --- | --- | --- | --- |
| **Breed** |  |  |  |  |
| Reference | Cobs | 1.0 |  | 0.02* |
|  | TB | 1.49 |  |  |
|  | WB | * |  |  |
|  | Welsh | * |  |  |
|  | ID | * |  |  |
|  | Other | 1.9 |  |  |
| **Sex** |  |  |  |  |
| Reference | Male | 1.0 |  |  |
|  | Female | 1.24 | 0.39-3.87 | 0.72 |
| **Anaesthetic Agent** |  |  |  |  |
| Reference | Sevoflurane | 1.0 |  |  |
|  | Isoflurane | 2.12 | 0.43-10.40 |  |
|  | Halothane | 2.75 | 0.33-23.27 | 0.42 |
| **Intra-operative Lidocaine** |  |  |  |  |
| Reference | Yes | 1.0 |  |  |
|  | No | 0.84 | 0.27-2.63 | 0.77 |
| **Period of Hypoxia** |  |  |  |  |
| Reference | Yes | 1.0 |  |  |
|  | No | 2.79 | 0.34-22.95 | 0.28 |
| **Post-operative Lidocaine** |  |  |  |  |
| Reference | Yes | 1.0 |  |  |
|  | No | 0.85 | 0.21-3.40 | 0.83 |
| **Post-operative Fluids** |  |  |  |  |
| Reference | Yes | 1.0 |  |  |
|  | No | 0.27 | 0.08-0.92 | 0.03* |
| **Type of Surgery** |  |  |  |  |
| Reference | Colic | 1.0 |  |  |
|  | Orthopaedic | 0.45 | 0.12-1.73 | 0.22* |
| **ASA score** |  |  |  |  |
| Reference | 1 | 1.0 |  | 0.32 |
|  | 2 | 0.48 | 0.04-5.67 |  |
|  | 3 | 0.25 | 0.03-2.43 |  |
|  | 4 | 0.17 | 0.02-1.50 |  |
|  | 5 | * | 0.00 | * |
| **Survival** |  |  |  |  |
| Reference | Yes | 1.0 |  |  |
|  | No | 3.61 | 1.03-12.61 | 0.05* |
