# Supplementary material for: The prevalence and nature of cardiac arrhythmias in horses following general anaesthesia and surgery
Source: Acta Vet Scand. 2011 Nov 23;53(1):62. doi: 10.1186/1751-0147-53-62 (PMC3269988; doi:10.1186/1751-0147-53-62)
Supplement: Additional file 9 — Outcome bradyarrhythmias Univariable Categorical Analyses.docx. [file 1751-0147-53-62-S9.DOC]

| **Continuous Variables**  Univariable binary logistic regression analyses of the continuous variables investigated in the study for their association with **sinus arrhythmia, atrio-ventricular block and sinus block**. | **Odds Ratio** | **95%Confidence interval** | **P value** |
| --- | --- | --- | --- |
| **Age (years)** | 0.94 | 0.84-1.04 | 0.21* |
| **Weight (Kg)** | 1.00 | 1.00-1.01 | 0.089 |
| **Pre-operative-op HR (bpm)** | 0.98 | 0.95-1.02 | 0.29 |
| **Pre-operative Na (mmol/l)** | 0.96 | 0.76-1.19 | 0.69 |
| **Pre-operative K (mmol/l)** | 2.02 | 0.47-8.70 | 0.33 |
| **Pre-operative Ca (mmol/l)** | 4.46 | 0.10-191.53 | 0.44 |
| **Pre-operative Cl(mmol/l)** | 1.01 | 0.86-1.19 | 0.86 |
| **Pre-operative COP (mmHg)** | 0.98 | 0.8-1.21 | 0.88 |
| **Post-operative Na T0 (mmol/l)** | 1.01 | 0.86-1.18 | 0.89 |
| **Post-operative K T0 (mmol/l)** | 1.36 | 0.38-4.86 | 0.63 |
| **Post-operative Ca T0 (mmol/l)** | 14.06 | 0.18-1095.43 | 0.23* |
| **Post-operative Cl T0 (mmol/l)** | 0.98 | 0.86-1.10 | 0.70 |
| **Post-operative COP T0**  **(mmHg)** | 0.99 | 0.79-1.25 | 0.94 |
| **Post-operative Na T12 (mmol/l)** | 1.01 | 0.85-1.21 | 0.88 |
| **Post-operative K T12 (mmol/l)** | 1.77 | 0.46-6.83 | 0.41 |
| **Post-operative Ca T12 (mmol/l)** | 3.52 | 0.05-228.11 | 0.55 |
| **Post-operative Cl T12 (mmol/l)** | 0.99 | 0.86-1.16 | 0.95 |
| **Post-operative COP T12 (mmHg)** | 1.07 | 0.91-1.26 | 0.41 |
| **Post-operative Na T24 (mmol/l)** | 1.01 | 0.83-1.22 | 0.95 |
| **Post-operative K T24 (mmol/l)** | 3.37 | 0.75-15.12 | 0.12* |
| **Post-operative Ca T24 (mmol/l)** | 129.30 | 1.13-14756.01 | 0.04* |
| **Post-operative Cl T24 (mmol/l)** | 0.99 | 0.82-1.20 | 0.90 |
| **Post-operative COP T24 (mmHg)** | 1.12 | 0.96-1.31 | 0.14* |
| **Post-operative HR0 (bpm)** | 0.95 | 0.92-0.99 | 0.005* |
| **Post- operative HR12 (bpm)** | 0.97 | 0.94-1.00 | 0.08* |
| **Post-operative HR24 (bpm)** | 0.98 | 0.94-1.01 | 0.16* |
