# Supplementary material for: The prevalence and nature of cardiac arrhythmias in horses following general anaesthesia and surgery
Source: Acta Vet Scand. 2011 Nov 23;53(1):62. doi: 10.1186/1751-0147-53-62 (PMC3269988; doi:10.1186/1751-0147-53-62)
Supplement: Additional file 10 — Outcome bradyarrhythmias Univariable Continuous Analyses.docx. [file 1751-0147-53-62-S10.DOC]

| **Continuous Variables**  Univariate ordinal logistic regression analyses of the continuous variables investigated in the study for their association with **supraventricular premature depolarisations**. | **Odds Ratio** | **95%Confidence Interval** | **P value** |
| --- | --- | --- | --- |
| **Age (years)** | 0.95 | 0.89-1.02 | 0.12* |
| **Weight (Kg)** | 1.00 | 1.00-1.00 | 0.44 |
| **Pre-operative Na (mmol/l)** | 1.01 | 0.89-1.16 | 0.83 |
| **Pre-operative K (mmol/l)** | 1.28 | 0.58-2.84 | 0.52 |
| **Pre-operative Ca (mmol/l)** | 6.80 | 0.65-70.71 | 0.10* |
| **Pre-operative Cl(mmol/l)** | 1.05 | 0.96-1.16 | 0.27 |
| **Pre-operative COP (mmHg)** | 1.06 | 0.93-1.21 | 0.37 |
| **Post-operative Na T0(mmol/l)** | 0.99 | 0.90-1.09 | 0.82 |
| **Post-operative K T0(mmol/l)** | 1.11 | 0.51-2.41 | 0.79 |
| **Post-operative Ca T0(mmol/l)** | 2.60 | 0.19-34-64 | 0.50 |
| **Post-operative Cl T0(mmol/l)** | 0.98 | 0.90-1.06 | 0.56 |
| **Post-operative COP T0 (mmHg)** | 1.03 | 0.89-1.19 | 0.68 |
| **Post-operative Na T12(mmol/l)** | 0.85 | 0.76-0.96 | 0.01* |
| **Post-operative K T12(mmol/l)** | 0.92 | 0.41-2.11 | 0.85 |
| **Post-operative Ca T12(mmol/l)** | 0.68 | 0.05-8.89 | 0.78 |
| **Post-operative Cl T12(mmol/l)** | 0.88 | 0.79-0.97 | 0.01* |
| **Post-operative COP T12 (mmHg)** | 1.01 | 0.91-1.12 | 0.88 |
| **Post-operative Na T24(mmol/l)** | 0.85 | 0.75-0.95 | 0.01* |
| **Post-operative K T24(mmol/l)** | 1.41 | 0.57-3.45 | 0.46 |
| **Post-operative Ca T24(mmol/l)** | 0.07 | 0.00-1.41 | 0.08* |
| **Post-operative Cl T24(mmol/l)** | 0.82 | 0.73-0.93 | 0.001* |
| **Post-operative COP T24 (mmHg)** | 1.02 | 0.92-1.12 | 0.73 |
| **Post-operative HR0 (bpm)** | 0.99 | 0.97-1.01 | 0.44 |
| **Post-operative HR12 (bpm)** | 1.01 | 0.99-1.03 | 0.40 |
| **Post-operative HR24 (bpm)** | 1.01 | 0.99-1.04 | 0.39 |
